# Analysis of Constraints on the Remote Application of Inverse Synthetic Aperture Laser Radar

**DOI:** 10.3390/s24113381

**Published:** 2024-05-24

**Authors:** Rui Gao, Lei Dong

**Affiliations:** 1 Changchun Institute of Optics, Fine Mechanics and Physics, Chinese Academy of Sciences, Changchun 130033, China; gaorui22@mails.ucas.ac.cn; 2 University of Chinese Academy of Sciences, Beijing 100049, China

**Keywords:** inverse synthetic aperture laser radar, action distance, laser power, coherence, optical path difference compensation

## Abstract

In order to achieve the remote application of inverse synthetic aperture laser radar for high resolution spatial situational awareness, it is essential to analyze the main factors that restrict its remote application. This study combines the range equation of inverse synthetic aperture lidar with the stimulated Brillouin threshold power equation to investigate the variation of laser transmitting power with distance. Additionally, by utilizing the excited Brillouin threshold power equation, laser linewidth formula, and pulse width characteristics of pulse signal, we examine the variation law of laser coherence that meets corresponding power requirements at different distances. The results indicate that a detection distance of 22 km and below can be achieved using continuous fiber lasers without compensation. Coherence compensation is necessary for distances between 22 km and 57 km. For distances ranging from 57 km to 3000 km, pulsed solid-state lasers are used to analyze coherence and conclude that imaging non-cooperative targets within this range is feasible. It is observed that coherence compensation is required from 57 km to 2179 km, becoming more challenging after 2000 km. Furthermore, pulsed solid-state lasers can still be utilized for imaging cooperative targets within a range of 2179–3273 km; however, coherence compensation remains necessary and becomes increasingly difficult. Finally, several coherent length compensation schemes are proposed in order to extend the imaging range of inverse synthetic aperture LiDAR to approximately 3000 km.

## 1. Introduction

With the advancement of space technology and its military applications, the scope of battlefields has expanded from land, sea, and air to include space. This expansion has broadened the territory of military activities and national security concerns. Space has gradually evolved into a strategic focal point for safeguarding national security and interests. The US military has expedited the integration of the space situational awareness network and initiated an integrated space situational awareness program. This program aims to establish space situational awareness capabilities for space operations by integrating mission clusters such as rocket launch detection, spacecraft monitoring and tracking, and space environment monitoring. Consequently, there is an urgent need for long-distance high-resolution detection and identification in all countries worldwide. This area also requires rapid development in China. However, traditional imaging methods have limitations when used over long distances. Therefore, it is necessary to explore new long-distance high-resolution imaging technologies. Inverse synthetic aperture Lidar (ISAL) possesses characteristics that make it suitable for this purpose—namely high-resolution (theoretically range-independent) and long-distance imaging capabilities. Luckey from the United States Naval Laboratory pointed out that with the current optical telescope caliber, synthetic aperture Lidar technology is the only method capable of achieving centimeter-level resolution imaging of targets located thousands of kilometers away [1].

SAL/ISAL technology is derived from SAR/ISAR [2,3,4,5], which extends the wavelength from radar microwave band to optical band to achieve higher resolution. In fact, as early as 1970, the applicability of the synthetic aperture principle was experimentally verified in the optical band in the United States. Between 1981 and 2018, Lincoln Laboratory invented and improved the “fire pool” lidar. Initially, the “fire pool” successfully obtained Doppler time-intensity images of the Agena D rocket booster 1350 km away, and the radar output peak power was increased to 45 kW. In 1992, the “fire pool” system reached a peak power output of 3.1 MW with the Main Oscillating Power Amplifier (MOPA). That same year, Lincoln Laboratory released the imaging results of the system from a laser Geodynamic satellite orbiting at an altitude of 5900 km. In this experiment, the signal bandwidth was 1 GHz, the pulse duration was 32 μs, and the pulse repetition rate was 8 Hz [1]. In 2013, the United States initiated the “long-range imaging Lidar” program, and in 2018, it launched the geosynchronous orbit ISAL [6]. In contrast, the latest literature in China reports that the detection of targets less than 10 km can be achieved [1], which still represents a gap compared to the international advanced level.

Therefore, this paper will study the constraints of the long-range application of inverse synthetic aperture LiDAR and attempt to extend its operating range to more than 100 km. Subject to the remote application scenario, the radar platform needs to emit very high power and good coherent beam (i.e., a single-frequency laser beam) interference to collect the target scattered echo. The better the coherence, the narrower the linewidth and the higher the image resolution [7]. According to literature research, the power and coherence of the laser are a pair of constraints [7,8], making it challenging to achieve high power and coherence simultaneously. For LiDAR long-distance applications, the linewidth is often required to be on the order of kilohertz (kHz). However, the narrower the laser linewidth, the more susceptible the laser power increase is to nonlinear effects, especially the stimulated Brillouin scattering (SBS), resulting in limited laser power. At the same time, the presence of nonlinear effects can also lead to the broadening of the laser linewidth, resulting in turn in reduced coherence [9]. Therefore, this paper will focus on studying the relationship between the two primary constraints of ISAL (space-based) remote applications—laser power and coherence—and try to provide a solution for the remote application of optical path difference compensation.

## 2. Basic Principle

### 2.1. ISAL (Space-Based) Remote Detection Principle

**Figure 1:**
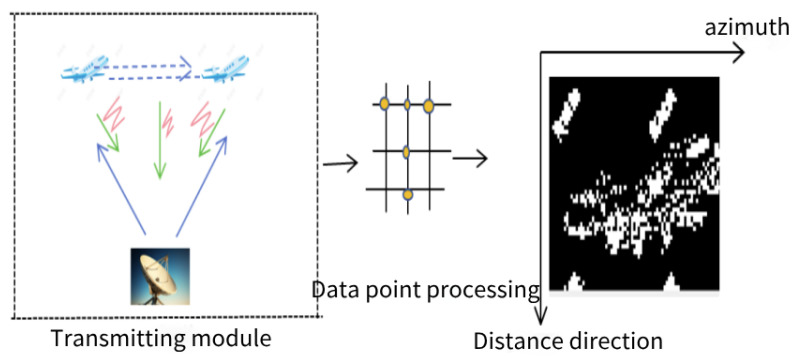
Schematic diagram of the basic principle of ISAL.

In inverse synthetic aperture LiDAR (ISAL), the radar is typically stationary, while the target is in motion. Therefore, ISAL is often used for ground-based radar to observe moving targets in the air or space-based radar platforms to observe small non-cooperative moving targets in distant space. In this paper, space-based radar (i.e., radar platform located on a satellite or an aircraft; specifically, including the NASA/LaRC spaceborne differential absorption radar developed by the United States, the CALIOP spaceborne lidar jointly developed by the United States and France, and so on) is used to explain the basic principle of ISAL, which involves the synthetic aperture principle. (1) Space-based radar sends waveform modulated signals, illuminates the target aircraft at a long distance, and then transmits the scattered echoes through the surface of the aircraft back to the radar. When ISAL is running, the pulse is sent and received at a fixed pulse repetition frequency, and the actual antenna takes the position of a simulated linear array antenna unit in sequence. The amplitude of the received signal from these unit antennas is combined with the relative phase of the transmitted signal needed to synthesize the received signal of an equivalent large-aperture antenna. (2) Application of Doppler effect: As the aircraft approaches the radar platform, the echo frequency increases to a maximum and then decreases as the aircraft moves away from the echo frequency. Based on the Doppler effect of relative motion, the movement in various dimensions can generate synthetic aperture images of varying dimensions, enabling the targeting of moving objects or objects with shaking characteristics. Three-dimensional imaging (i.e., frontal, lateral, and top-down views) is achieved by maintaining the LiDAR in a fixed position, thereby improving concealment in critical situations like on the battlefield. (3) Imaging principle: The radar interferes with the echo of each time period and the reference light, reflecting the information of light intensity and amplitude through the phase information. It compresses the range and azimuth data and then estimates the information of the moving target. The specific schematic diagram is shown in Figure 1.

Therefore, ISAL relies on the Doppler effect of target motion to transmit and receive signals, enabling the establishment of synthetic aperture and obtaining a two-dimensional image of the target. The formulas for distance and azimuth resolution of the image are shown in (1) and (2), respectively [10]. According to the formula, the resolution is independent of the distance.

(1) $$\rho_{r}=\frac{c}{2B}$$

(2) $$\rho_{a}=\frac{\lambda }{2\Delta \theta }$$

In Equation (1), c is the speed of light in vacuum, B is the bandwidth of the transmitted signal, and $\rho_{r}$ is the range resolution of ISAL.

In Equation (2), λ is the wavelength of the signal light, Δθ is the Angle of the target relative to ISAL, and $\rho_{a}$ is the azimuth resolution of ISAL.

### 2.2. Coherent Detection Principle

**Figure 2:**
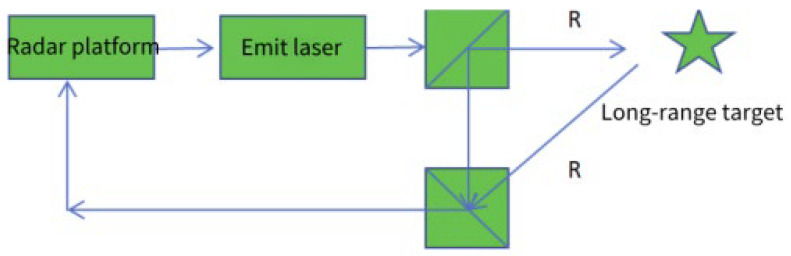
Schematic diagram of coherent detection principle.

Coherent detection is an effective method for ISAL to achieve accurate detection of distant targets. The radar target interferes with the reference light through the echo of each time period, and the information about light intensity and amplitude is reflected through the phase information. First, the radar platform emits a laser signal and splits the outgoing light into two paths using a beam splitter. One path continues to illuminate the distant target; one way remains stationary and interferes with the scattering echo of the target. This interference occurs through the beam splitter coupling with the delay control. Subsequently, the signal is returned to the radar platform for digital signal processing. At the same time, because the receiving phase originates from the interference of the laser beam, the clutter noise does not interfere. This is equivalent to automatically suppressing background noise, allowing the system to operate not only at night but also during the day. The schematic diagram of the coherent detection principle is shown in Figure 2.

## 3. Simulation Analysis of the Core Influencing Factors on Remote Applications

As can be seen from the above introduction, the application of space-based inverse synthetic aperture lidar in long-distance imaging is limited by two core factors: (1) The laser source transmission power must be large enough to ensure that the target echo can be received by the detector. (2) The laser coherence must be sufficient (mainly referring to good time coherence, i.e., a large enough coherence length) to meet the interference detection requirements of the target echo and the local reference wave. Therefore, the two main influencing factors of ISAL (space-based) remote sensing applications, laser power and laser coherence, are analyzed below.

### 3.1. Analysis of Influencing Factors of Laser Power on Remote Applications

Firstly, based on the traditional laser action distance equation, combined with the parameter selection in the ISAL (space-based) application process, the laser emission power required for long distances (10–3000 km) was used. Through literature research, the ISAL action distance equation [11] is presented in Equation (3).

(3) $$P_{out}=\frac{SNR\cdot 4\pi \cdot \Omega \cdot F\cdot h\cdot f\cdot R^{4}}{\gamma_{\mathrm{sys}}\cdot \gamma_{\mathrm{ato}}\cdot G\cdot \omega \cdot S\cdot T_{p}}$$

In Equation (3), h is Planck’s constant, R is the detection distance, and f is the frequency of the 1064 nm laser. The meanings and values of other parameters in the formula are shown in Table 1.

#### 3.1.1. Analysis of Influence Factors on Power of Continuous Fiber Laser

**Figure 3:**
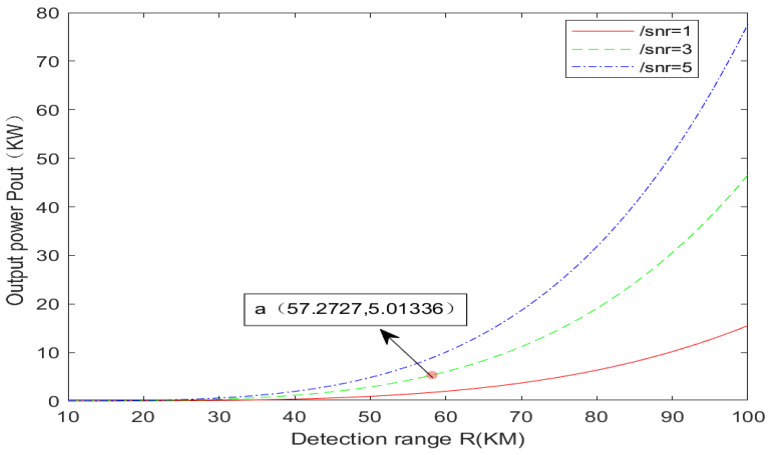
Power required for 10–100 km ISAL laser emission.

MATLAB R2012a was used to analyze the power required for ISAL laser emission at 10 km to 100 km, as shown in Figure 3. There are three curves in Figure 1, representing the signal-to-noise ratio of images under three different conditions of 1 dB, 3 dB, and 5 dB. To reduce the demand for laser emission power, a 3 dB curve is chosen for analysis. Through an investigation of the latest advancements in domestic literature, it is evident that the current domestic 1064 nm high-power narrow line-width continuous fiber laser can achieve a maximum of 5.07 kW [12]. This corresponds to point A in Figure 3, indicating that the power requirement of approximately 57 km is met. In other words, the target within 57 km is fully satisfied with the continuous wave (CW) laser power, while the target beyond 57 km can only rely on the peak power emitted by the pulsed laser to meet the detection conditions.

#### 3.1.2. Analysis of Influence Factors of Pulsed Laser Power

**Figure 4:**
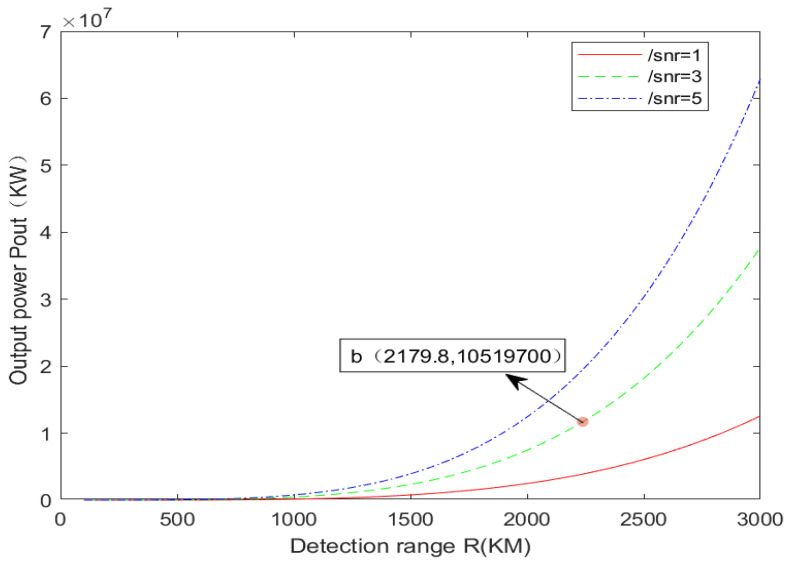
100–3000 km ISAL power required for laser emission.

Then, the further distance (100–3000 km) is simulated and analyzed, and the simulation results are shown in Figure 4. Similarly, this paper selects the curve when SNR is 3 dB for analysis. By investigating the recent advancements in high-power pulse lasers, it is evident that the current output energy of the pulse laser can reach 105 J [13] through a five-stage amplification structure. The corresponding peak power is approximately 10.5 GW, with a pulse width of 10 ns and a pulse repetition frequency of 10 Hz. This point roughly corresponds to point b in Figure 4. In other words, the detection of any non-cooperative target within 2179 km can be achieved by continuously increasing the transmitting power. If you aim to achieve the detection of further targets based on this approach, for cooperative targets, it might be feasible to utilize targets equipped with multiple angled mirrors to minimize the scattering angle of the laser beam reflected from the target surface. In the case of a cooperative target, the echo divergence angle is approximately equal to the incident light divergence angle. This helps to reduce the scattering area, indirectly decrease the need for transmitting power, and enables the detection of more distant targets. For example, when the emission angle of the incident light is π/4, the solid angle of the target scattering is approximately doubled, that is, π^2^/16, which decreases π/16 times compared with Ω = π in Table 1. It can be seen from P_out_ ∝ Ω ∝ R^4^ that the power decreases by π/16 times, and the detection distance correspondingly increases by (16/π)^1/4^ times. That is, it can detect a maximum distance of 2179 × (16/π)^1/4^ ≈ 3273 km.

### 3.2. Analysis of Influencing Factors of Coherence on Remote Applications

**Figure 5:**
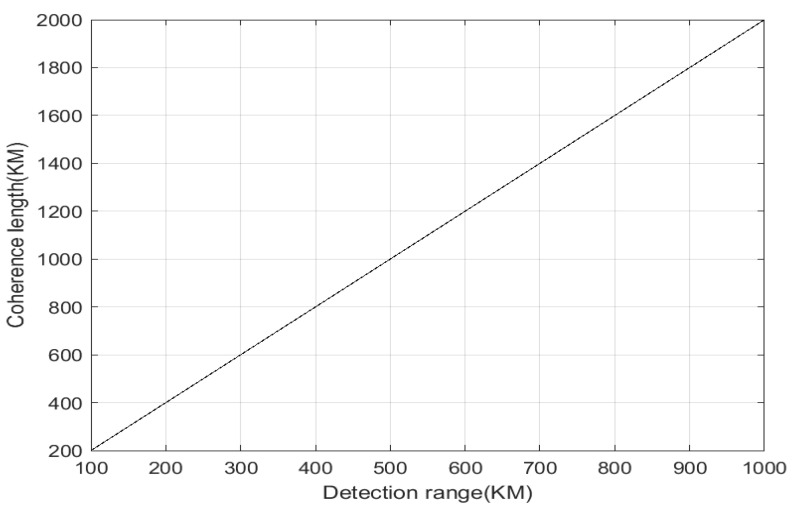
The relationship between laser coherence length and detection distance.

For the inverse synthetic aperture LiDAR, due to the coherent detection system, when achieving remote target acquisition and imaging, it is essential to adhere to both the transmission power limitations and the coherence requirements. According to literature research, some articles indicate that when the echo interferes with the local oscillator signal delay light, it needs to be equal to the detection distance in the coherence length order [7], and some documents even suggest that the detection distance should be doubled [14]. For the rigor of this paper, twice the detection distance is adopted, as illustrated in Figure 5.

**Figure 6:**
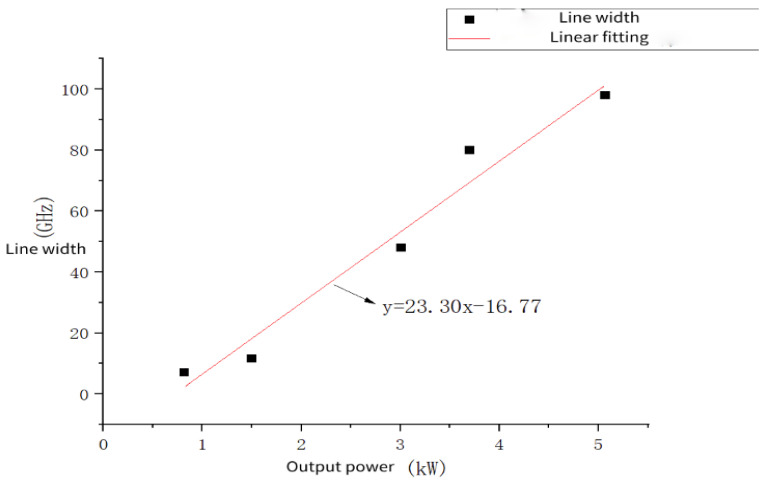
Linear fitting of 1064 nm fiber laser power and linewidth.

As mentioned above, in order to achieve the detection of long-distance targets, two core conditions need to be met for the laser: one is to have a large transmission power, and the other is to have good coherence. The index used to measure the coherence of a laser is its spectral linewidth, commonly referred to as the linewidth. The narrower the linewidth, the better the coherence. From a simple linear fitting of five groups of power and linewidth data points [9] in the research progress of 1064 nm fiber lasers, the results are shown in Figure 6 (the fitting only describes the rough relationship between laser power and coherence. In reality, it is not an ideal linear relationship. Usually, the linewidth of the laser is related to power, pulse width, loss, and other factors). It is not difficult to find that the power and coherence of the laser are mutually restricted. In other words, an increase in power will cause the laser’s linewidth to widen, resulting in decreased coherence and the inability to achieve interference.

According to the principal diagram of inverse synthetic aperture LiDAR and the characteristics of a 1064 nm single-frequency fiber laser with a narrow linewidth and low noise, a continuous fiber laser with a wavelength of 1064 nm is selected for specific analysis for operating distances less than 57 km. For over 57 km, pulsed solid-state laser analysis is utilized.

#### 3.2.1. Analysis of Factors Affecting Coherence of Continuous Fiber Lasers

High-power fiber lasers typically utilize a coherent seed source for multistage amplification, based on a Master Oscillator Power Amplifier (MOPA) structure. It is known by the coherence of time.

(4) $$\Delta \nu_{s}\times \Delta t\approx 1$$

In Equation (4), Δν_s_ is the linewidth, and Δt is the coherence time.

The relationship between laser coherence length and linewidth can be determined by multiplying both sides by the speed of light simultaneously, as shown in Formula (5).

(5) $$L_{c}=\frac{c}{\Delta \nu_{s}}$$

From Equation (5), it can be seen that in order to detect a target at 100 km, the coherence length $L_{c}$ must be 200 km, which implies that the linewidth of the laser must be 1.5 kHz. Therefore, to detect targets beyond 100 km, the laser’s linewidth needs to be in the order of kHz.

In addition, because the power required for remote target detection has reached the order of kilowatts (kW) or even gigawatts (GW), which is much higher than the power level that can be achieved in China at present, the laser will also produce nonlinear effects while achieving high power. For example, SBS (stimulated Brillouin scattering) is more likely to occur when the linewidth is narrow, SRS (stimulated Raman scattering) is more likely to occur when the linewidth is wide, and LMI (lateral mode instability) is more likely to occur when the optical fiber core mode field diameter is too large [9]. Therefore, a single-mode fiber with a length of 1 m, a mode field diameter of 30 μm, and an attenuation coefficient of 0.4 dB/km are selected [9]. Based on the single-frequency narrow linewidth characteristics of ISAL lasers, fiber lasers are more susceptible to SBS. Through literature research, the relationship between the threshold power of SBS and the linewidth of the laser [9] is depicted in Equation (6).

(6) $$P_{SBS}\approx \frac{21K*A_{eff}}{g_{B}*L_{eff}}\left(1+\frac{\Delta \nu_{s}}{\Delta \nu_{B}}\right)In\left(G\right)$$

Parameters and values in the formula are shown in Table 2.

**Figure 7:**
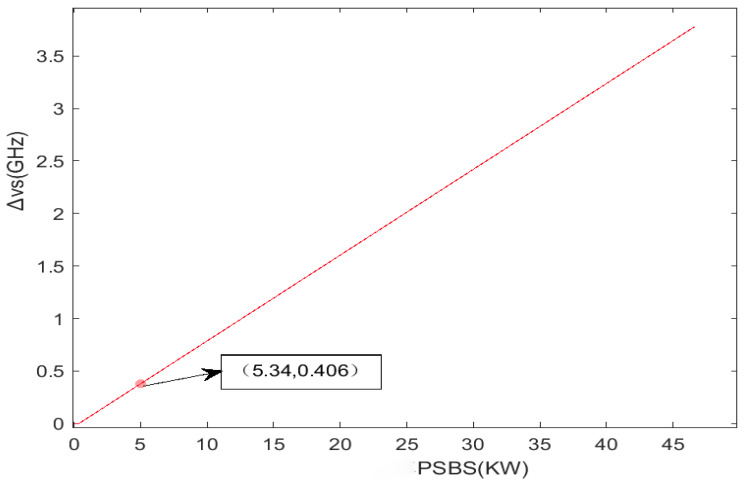
The relationship between PSBS and laser linewidth $\Delta \nu_{s}$.

According to Equation (6), it can be found that the threshold power PSBS of stimulated Brillouin scattering is positively correlated with the laser linewidth Δν_s_. Laser transmission power P_out_ can be emitted normally only after overcoming SBS threshold power, that is, P_out_ = P_SBS_; Further, the straight line between P_SBS_ and laser linewidth Δν_s_ at 10–100 km is simulated by Equation (6), as shown in Figure 7. It can be found that when the output power is 5 KW, the laser linewidth Δν_s_ is roughly in the order of GHz.

**Figure 8:**
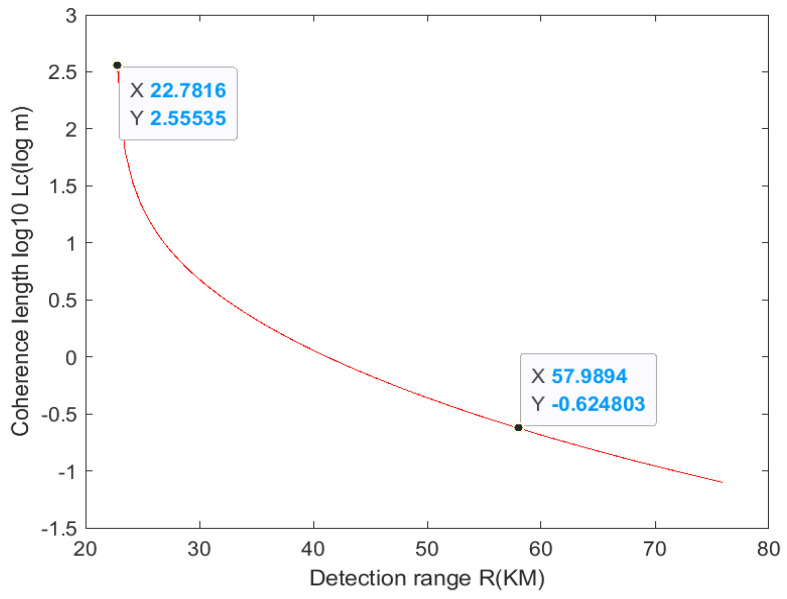
The relationship between 10−100 km distance and laser coherence length.

After combining Formulas (3), (5), and (6), the laser coherence length under different detection distances (10–100 km) is obtained, as shown in Figure 8.

As can be seen from Figure 8, when the detection distance is less than 22 km (the discontinuity point when $\Delta \nu_{s}$ is 0), the coherence length is not restricted by the transmission power and fully meets the requirements of long-distance imaging. Then the coherence decreases sharply, and the coherence length is 10^−0.624^ = 0.23 m at 57 km. Combined with the above conditions, ensuring that the power of the CW fiber laser is fully satisfied within a 57 km range, it can be concluded that the CW fiber laser can be utilized for long-distance detection within the 10–22 km range without compensation for long-distance detection. The laser coherence length of 22 km to 57 km needs to be compensated for.

#### 3.2.2. Analysis of Influencing Factors of Coherence of Pulsed Laser

**Figure 9:**
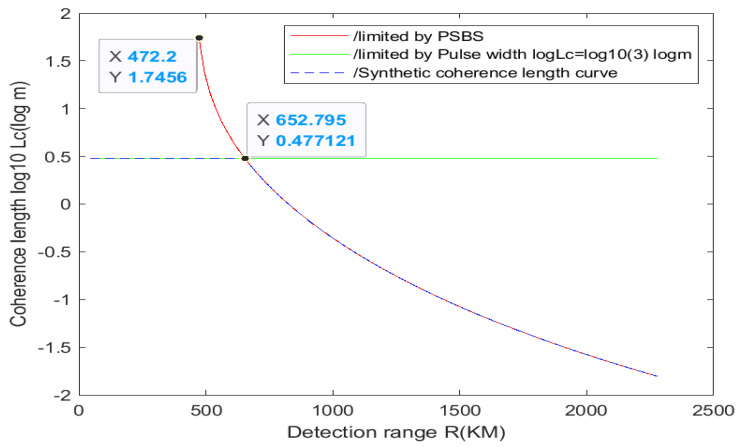
The relationship between 57–3000 km distance and laser coherence length.

Since the transmission power of a continuous fiber laser can only reach 57 km, the coherence change beyond this distance is analyzed using pulsed laser. Pulsed solid-state lasers can achieve longer-distance coherent detection while maintaining high transmission power. In terms of parameter selection, the diameter of the mode field is changed from 30 μm to 10 mm, while the other parameters remain the same as in Table 2. Pulsed lasers are typically very powerful but have a very small signal pulse width. The pulse width of the pulse laser cannot be too small, as a very small pulse width can easily cause signal interference. For instance, if the pulse width is 10 ns, its coherence length would be 3 × 10^8^ m/s × 10 ns = 3 m. Taking into account the analysis of SBS above, the coherence length at this point should be the minimum value resulting from the combination of the two factors. The coherence length ranges from 57 km to 3000 km at various distances, and the simulation results are depicted in Figure 9.

It is not difficult to find from Figure 9 that the coherence length is 55 m at 472 km (the discontinuity point when $\Delta \nu_{s}$ is 0) and 3 m at the intersection of the two curves at 652 km. The coherence length before the intersection is mainly limited by pulse width, and after the intersection, it is mainly limited by PSBS. The coherence length approaches 0 at 2000 km. Therefore, the use of pulsed solid-state lasers for distances ranging from 57 km to 3000 km requires coherent compensation. This is because the optical path difference is less than the actual coherence length needed to achieve heterodyne interference detection. When the lower limit of compensation accuracy equals the actual coherence length, it just meets the interference conditions. Additionally, the compensation accuracy is generally 1/10 of the coherence length.

In short, (1) continuous fiber lasers can be used for detection distances below 22 km without coherence compensation. Coherence compensation is required for 22 km to 57 km. (2) The detection range of 57 km to 2179 km can be achieved through pulsed solid-state laser imaging of non-cooperative targets. The coherence compensation is required from 57 km to 2179 km, and it becomes more challenging after 1000 km. For cooperative target imaging, pulsed solid-state lasers ranging from 2179 km to 3273 km can still be utilized. Coherence compensation remains necessary, albeit more challenging than before.

## 4. Coherence Compensation Scheme

### 4.1. Large-Travel Fast-Variable Fiber Delay Line Topology Based on Magneto-Optic Switch

**Figure 10:**
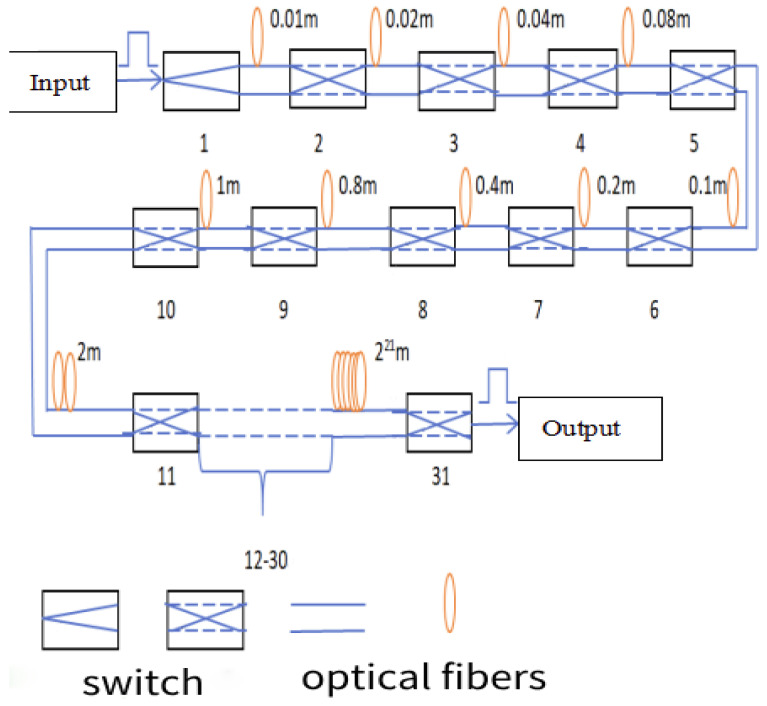
A schematic diagram of a large-stroke fast-variable fiber delay line topology based on magneto-optic switching.

Given the coherent conditions necessary for remote target detection, the laser’s coherence is insufficient for interference, necessitating timely optical compensation. The first consideration is the design of the optical fiber delay line, utilizing a magneto-optical control switch. Its response speed can range from about 10 μs to 20 μs, with minimal insertion loss. A multi-level topology can be employed to cater to varying coherent length differences for different distances, ensuring different compensation accuracies. By controlling different switches simultaneously, delay compensation can be achieved effectively. The structural diagram of the large-stroke fast-variable fiber delay line topology based on magneto-optical switching is illustrated in Figure 10 [15].

Table 3 below is a random example to illustrate the preceding sequence of individual distance switches.

Therefore, the technology can achieve distance compensation ranging from 1 m to 4,194,303 m by controlling various switches simultaneously. The adjustment interval can be as precise as 1 m, with a maximum compensation accuracy of 1 m and a minimum accuracy of 0.01 m.

### 4.2. Other Coherence Compensation Methods

In addition to the coherence compensation mentioned above using topology-based segmented fiber delay lines, researchers have also proposed other compensation methods. Li Daojing et al. proposed using local oscillator digital delay technology [16] to determine the delay amount. The principle involves establishing a reference channel for the local oscillator signal after splitting. Subsequently, the optical phase change of the incoming local oscillator signal is estimated through coherent detection. This process replaces digital signal processing to compensate for the digital delay of the echo signal in real time. This method can also be tried for experimental verification.

In addition, the experimental verification of direct view SAL [17] technology based on deviation detection by Liu Liren’s team shows that this method is a technology with a theoretical optical delay of 0. Therefore, it can avoid the issues related to poor laser coherence or a deficient optical system. Its principle is to use two beams of coaxial, concentric, and polarized orthogonal light emitted simultaneously for auto differential reception. This ensures that the optical path difference is always 0, and the imaging data processing of the subsequent echo is consistent with SAL.

## 5. Conclusions

By comparing the development of long-distance ISAL in China and abroad, this paper highlights the importance of researching long-distance ISAL. Based on the basic principle of ISAL and the principle of coherent detection, a simulation is conducted using the ISAL (space-based) distance equation and SBS threshold power equation. The constraints in the remote scenario are studied with the given parameters, leading to the following conclusions:

(1) The remote detection performance of fiber lasers is being studied. The simulation results show that the continuous fiber laser can be used to detect operating distances of 22 km and below without compensation. Between 22 km and 57 km, coherent compensation is required.
(2) By choosing a pulsed solid-state laser to adjust the coherence length within the range of 57 km to 3000 km, while considering the limited pulse width and SBS threshold power, it is determined that coherence compensation is necessary for distances spanning from 57 km to 3000 km. In addition, it is proposed that an angle reflector (cooperative target) can be used to reduce the power demand for detection, thus enabling longer distance detection. The text explains the difficulty of compensation for cooperative and non-cooperative goals, respectively.
(3) Some mature schemes for optical coherence compensation are presented.

## Figures and Tables

**Table 1:** Parameter selection of ISAL operating distance equation.

Physical Parameter	Physical Meaning	Numerical Value
SNR	Image signal-to-noise ratio	1/3/5 dB
Ω	Target backscatter solid angle	π
F	Electronic noise figure	3 dB
$\gamma_{\mathrm{ato}}$	Atmospheric transmittance	1
$\gamma_{\mathrm{sys}}$	System loss	0.18
$T_{p}$	Pulse width	5 μs
G	Transmission gain	33.5 × 10^6^
ω	Target scattering area	2.5 × 10^−4^ m^2^
S	Effective receiving area of the telescope	4π m^2^

**Table 2:** Parameter selection of SBS threshold power equation within 100 km.

Physical Parameter	Physical Meaning	Numerical Value
*K*	Polarization-dependent factor	1
*G*	Brillouin gain coefficient	25.9686
*A_eff_*	Optical fiber mode field area	7.07 × 10^−10^ m^2^
*g_B_*	Peak Brillouin gain	5 × 10^−11^ m/W
∆*ν_B_*	Brillouin scattered light bandwidth	30 MHz
*L_eff_*	Effective length of fiber	0.998 m

**Table 3:** The preceding sequence of individual distance switches.

Detection Range	Compensating Distance	Compensation Accuracy	Simultaneous Switch
57 km	114 km	2	26, 25, 23, 22, 21, 20, 18, 16, 14
652 km	1304 km	6	30, 27, 26, 25, 24, 23, 20, 18, 17, 16
1000 km	2000 km	2	30, 29, 28, 27, 25, 20, 17

## Data Availability

The data presented in this study are available on request from the corresponding author. The data are not publicly available due to technological security.
